# PRDM16 deficiency in vascular smooth muscle cells aggravates abdominal aortic aneurysm

**DOI:** 10.1172/jci.insight.167041

**Published:** 2023-06-08

**Authors:** Zhenguo Wang, Xiangjie Zhao, Guizhen Zhao, Yanhong Guo, Haocheng Lu, Wenjuan Mu, Juan Zhong, Minerva Garcia-Barrio, Jifeng Zhang, Y. Eugene Chen, Lin Chang

**Affiliations:** 1 Department of Internal Medicine, Cardiovascular Center, University of Michigan Medical Center, Ann Arbor, Michigan, USA.; 2 Key Laboratory of Animal Cellular and Genetics, Engineering of Heilongjiang Province, College of Life Science, Northeast Agricultural University, Harbin, P.R. China.; 3 Department of Pharmacology, Southern University of Science and Technology, Shenzhen, P.R. China.

**Keywords:** Autoimmunity, Vascular Biology, Apoptosis, Mouse models

## Abstract

Abdominal aortic aneurysm (AAA) is usually asymptomatic until life-threatening complications occur, predominantly involving aortic rupture. Currently, no drug-based treatments are available, primarily due to limited understanding of AAA pathogenesis. The transcriptional regulator PR domain–containing protein 16 (PRDM16) is highly expressed in the aorta, but its functions in the aorta are largely unknown. By RNA-seq analysis, we found that vascular smooth muscle cell–specific (VSMC-specific) *Prdm16*-knockout (*Prdm16*^SMKO^) mice already showed extensive changes in the expression of genes associated with extracellular matrix (ECM) remodeling and inflammation in the abdominal aorta under normal housing conditions without any pathological stimuli. Human AAA lesions displayed lower PRDM16 expression. Periadventitial elastase application to the suprarenal region of the abdominal aorta aggravated AAA formation in *Prdm16*^SMKO^ mice. During AAA development, VSMCs undergo apoptosis because of both intrinsic and environmental changes, including inflammation and ECM remodeling. *Prdm16* deficiency promoted inflammation and apoptosis in VSMCs. A disintegrin and metalloproteinase 12 (ADAM12) is a gelatinase that can degrade various ECMs. We found that ADAM12 is a target of transcriptional repression by PRDM16. *Adam12* knockdown reversed VSMC apoptosis induced by *Prdm16* deficiency. Our study demonstrated that PRDM16 deficiency in VSMCs promoted ADAM12 expression and aggravates AAA formation, which may provide potential targets for AAA treatment.

## Graphical abstract

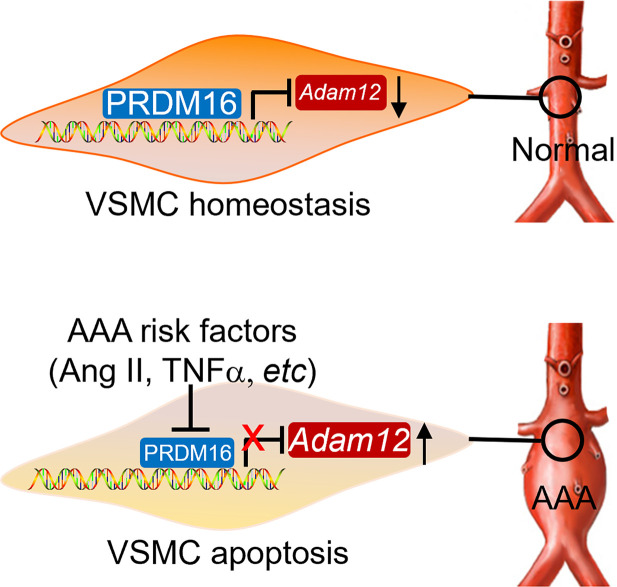

## Introduction

Abdominal aortic aneurysm (AAA) is a permanent and irreversible dilation of the abdominal aorta (1, 2). Patients with AAA are often asymptomatic but at high risk of rupture, the main complication of AAA, with mortality of 85% to 90% (3). Smoking, male sex, older age, family history, hypertension, and atherosclerosis are recognized as primary risk factors for AAA (1, 2). The hallmarks of AAA include extracellular matrix (ECM) fragmentation/degradation, vascular smooth muscle cell (VSMC) death, increased oxidative stress, and immune cell infiltration in the aorta (4, 5). Open surgery and endovascular aortic repair are conventional clinical interventions currently available for AAA treatment but are only considered for patients with large AAA (≥55 mm), while no treatment options are available for patients with smaller, asymptomatic AAA. Currently, there are no effective pharmacological treatments for AAA. Therefore, increased understanding of AAA pathogenesis could guide the development of alternative treatments for AAA.

PR domain–containing protein 16 (PRDM16) is a regulator of transcription that governs gene expression by cooperating with other transcription factors. It is involved in acute myeloid leukemia (6, 7), brown and beige adipocyte fate determination and functional regulation (8, 9), palatogenesis (10), hematopoiesis (11), and cardiac development and function (12–14). However, the role of PRDM16 in the vasculature is less explored. PRDM16 has been shown to be expressed in the arterioles of mouse retina, where it marks a single retinal ganglion cell subtype and controls arterial vessel development and/or function (15). A recent study revealed that PRDM16 is highly expressed in arterial endothelial cells and smooth muscle cells (SMCs), and that PRDM16 in endothelial cells but not SMCs is indispensable for normal arterial blood flow recovery in peripheral artery disease (16). Despite the high expression of PRDM16 in the aorta, there is a lack of understanding of its function in VSMCs. Therefore, we sought to investigate the function of PRDM16 in the aorta, specifically in the VSMCs, and in association with AAA formation.

In the present study, we found that VSMC-specific *Prdm16*-KO mice exhibited aggravated AAA formation in an elastase application model. By using primary VSMCs and A7r5 cells, we found that PRDM16 deficiency resulted in exacerbated apoptosis. We further identified a disintegrin and metalloproteinase 12 (ADAM12) as a transcriptional target of repression by PRDM16, and knockdown of *Adam12* reversed apoptosis induced by PRDM16 deficiency.

## Results

### PRDM16 deficiency in VSMCs induces remodeling of abdominal aorta in mice.

PRDM16 is highly expressed in aortic and coronary tissues (Supplemental Figure 1; supplemental material available online with this article; https://doi.org/10.1172/jci.insight.167041DS1), and is emerging as a locus that is associated with various manifestations of cardiovascular diseases. For instance, the phenome-wide association (PheWAS) plot in the Common Metabolic Diseases Knowledge Portal (https://hugeamp.org/ Accessed March 15, 2023.) reveals that single-nucleotide polymorphisms (SNPs) of *PRDM16* are significantly associated with hypertension and cholesterol metabolism (rs2493296), coronary artery disease (rs7413494), and any stroke (rs2455129). Additionally, 2 separate genome-wide association studies have demonstrated that multiple SNPs within *PRDM16* (rs72633335, rs6670123, rs2493288, rs2493290, rs2493291, rs2493292, rs2493296, and rs2493298) are associated with a higher risk of developing coronary artery disease (17) and hypertension (18) within certain populations (Supplemental Figure 2). Therefore, we propose that PRDM16 may play important roles in vascular biology.

**Figure 1:**
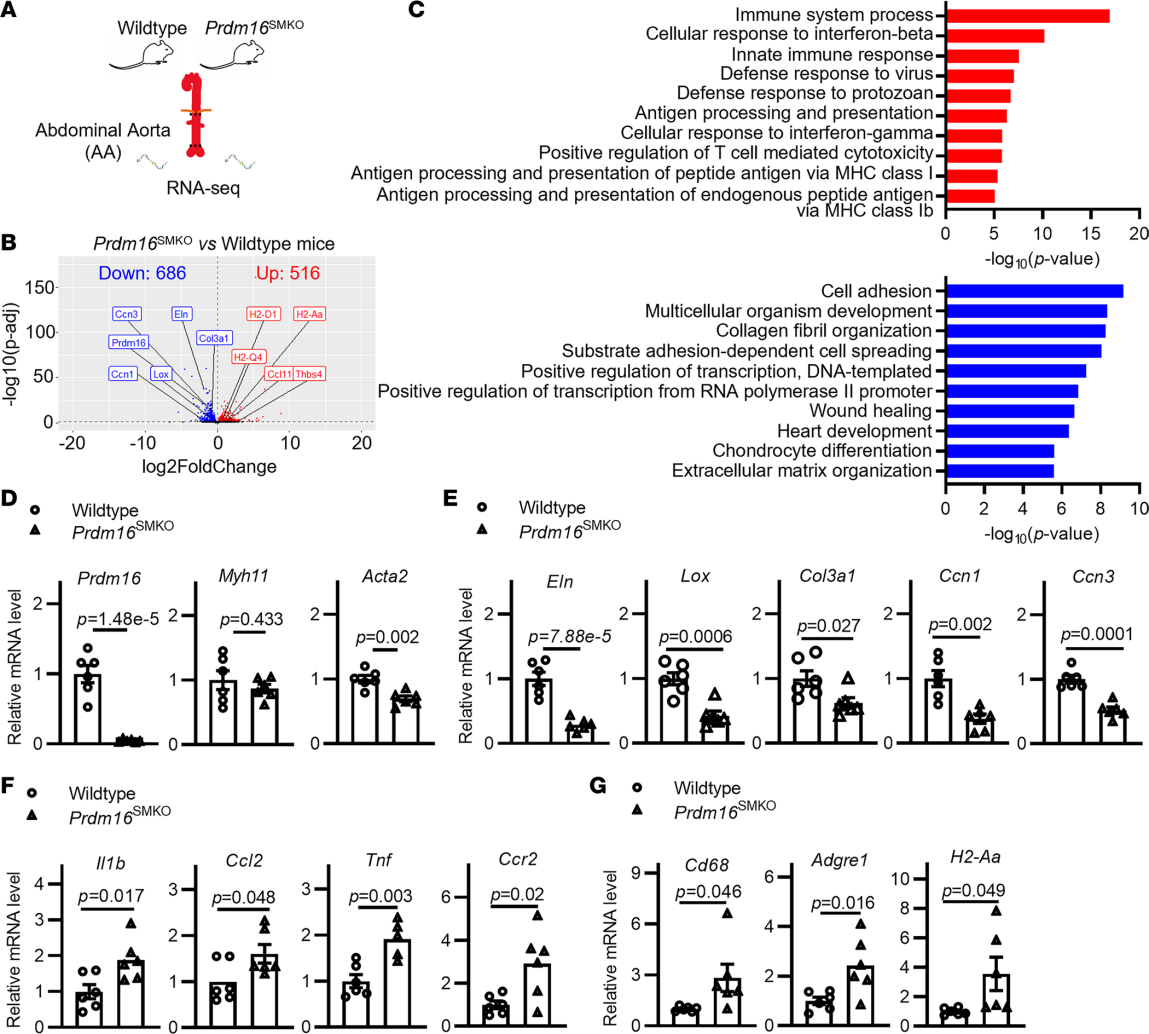
RNA-seq reveals remodeling of abdominal aorta from VSMC-specific *Prdm16*-knockout mice. (**A**) RNA-seq analysis of the abdominal aorta from VSMC-specific *Prdm16*-knockout mice (*Prdm16*^SMKO^) and wild-type control mice (*Myh11*-CreER^T2^) (*n* = 3). (**B**) Volcano plot of the differentially expressed genes (DEGs) with *P_adj_* < 0.05; upregulated DEGs are highlighted in red, and downregulated DEGs are highlighted in blue. (**C**) The DEGs were analyzed for gene ontology biological process (GO_BP) enrichment using DAVID, and the top 10 significantly enriched terms are shown. Red bars and blue bars indicate GO_BP results from upregulated DEGs and downregulated DEGs, respectively. (**D**) qPCR verification of *Prdm16* and qPCR verification of VSMC contractile genes, *Myh11* (myosin heavy chain 11) and *Acta2* (α2-actin). (**E**) qPCR verification of ECM-related genes *Eln* (elastin), *Lox* (lysyl oxidase), *Col3a1* [collagen α-1(III) chain], and *Ccn1* (cellular communication network factor 1). (**F**) qPCR verification of inflammation-related genes *Il1b* (IL-1β), *Ccl2* (C-C motif chemokine 2), *Tnf* (TNF-α), and *Ccr2* (C-C chemokine receptor type 2). (**G**) qPCR verification of immune cell marker genes *Cd68* (macrosialin), *Adgre1* (cell surface glycoprotein F4/80), and *H2-Aa* (H-2 class II histocompatibility antigen, A-B α chain). The gene expression was normalized to *Gapdh*. Data are presented as mean ± SEM, *n* = 6. *P* values were calculated using 2-tailed Student’s *t* test (**D**–**G**).

To begin addressing the role of PRDM16 in the vasculature, we generated VSMC-specific *Prdm16*-KO mice (*Prdm16^fl/fl^* *Myh11*-CreER^T2^, hereafter *Prdm16*^SMKO^) by crossbreeding *Prdm16^fl/fl^* mice with *Myh11*-CreER^T2^ mice. The PRDM16-associated gene expression profile was assessed by RNA sequencing (RNA-seq) analysis of abdominal aortas from adult *Prdm16*^SMKO^ and wild-type control mice (Figure 1A). The analysis of differentially expressed genes (DEGs) identified 516 upregulated genes and 686 downregulated genes in *Prdm16*^SMKO^ mice compared with control mice (Figure 1B). Gene ontology (GO) analysis revealed that the upregulated genes were significantly enriched in the biological processes related to inflammation, including immune system process, innate immune response, cellular response to IFN-β/γ, and antigen processing and presentation. The downregulated genes were significantly enriched in the GO terms of cell adhesion, collagen fibril organization, and ECM organization (Figure 1C). These data suggest that the abdominal aortas of *Prdm16*^SMKO^ mice exhibit increased inflammation and ECM remodeling. This was further supported by quantitative real-time PCR (qPCR) analysis, which revealed that the mRNA levels of the VSMC marker gene *Acta2* (Figure 1D) and ECM-related genes, including *Eln*, *Lox*, *Col3α1*, and *Ccn1* (Figure 1E), were significantly decreased, while the proinflammatory genes, including *Il1b*, *Ccl2*, and *Ccr2* (Figure 1F), and the macrophage marker genes *Cd68* and *Adgre1* (Figure 1G), were significantly increased in the abdominal aorta of *Prdm16*^SMKO^ mice compared with those of control. These data indicate that PRDM16 deficiency induces ECM remodeling and inflammation in the abdominal aorta of mice, which are key features of many vascular diseases, including AAA (4). Next, we sought to investigate the role of PRDM16 in AAA formation.

### PRDM16 expression is reduced in human AAA lesions.

**Figure 2:**
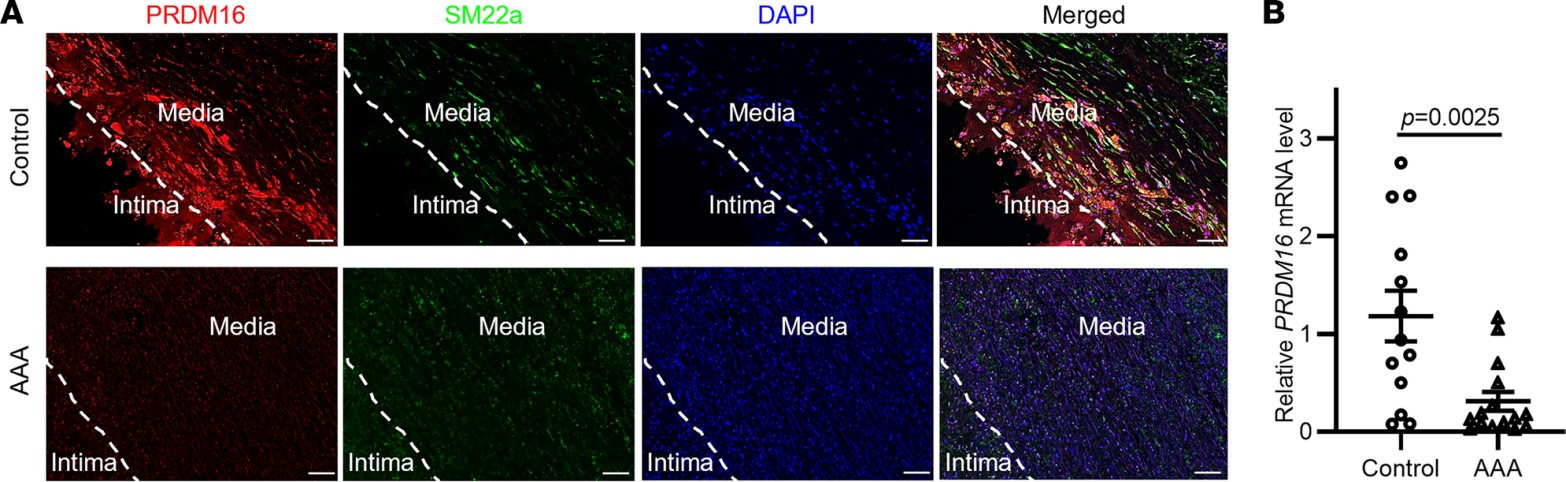
PRDM16 expression is reduced in human abdominal aortic aneurysm (AAA) lesions. (**A**) Representative immunofluorescent staining of PRDM16 (red), smooth muscle protein 22α (SM22α, green), and nuclei (4′,6-diamidino-2-phenylindole [DAPI], blue) in human AAA lesions and normal aortic tissues. Scale bars: 100 μm. (**B**) *PRDM16* mRNA expression was determined by qPCR in human AAA samples (*n* = 15) and control tissues (*n* = 13). The gene expression was normalized to *GAPDH*, and data are presented as mean ± SEM. *P* value was calculated using 2-tailed Student’s *t* test.

To investigate whether PRDM16 is involved in AAA pathology, we determined PRDM16 protein abundance in human AAA lesions by immunofluorescent staining (Figure 2A). We found that human AAA lesions displayed decreased PRDM16 expression in the VSMCs of the aortic medial layer (SM22α serves as VSMC marker). We also compared *PRDM16* mRNA expression in human AAA samples and normal abdominal aortic tissues (patient information in Supplemental Table 1) using qPCR and confirmed that *PRDM16* mRNA expression was significantly reduced in human AAA lesions (Figure 2B). Moreover, we analyzed the expression of *Prdm16* in mouse VSMCs from 3 single-cell RNA-seq data sets (GSE152583, elastase-induced AAA, ref. 19; GSE164678, CaCl_2_-induced AAA, ref. 20; and PRJCA006049, angiotensin II–induced AAA, ref. 21), and the results showed significantly decreased expression of *Prdm16* in VSMCs from AAA mice compared with control mice (Supplemental Figure 3).

### PRDM16 deficiency in VSMCs aggravates elastase-induced AAA in mice.

**Figure 3:**
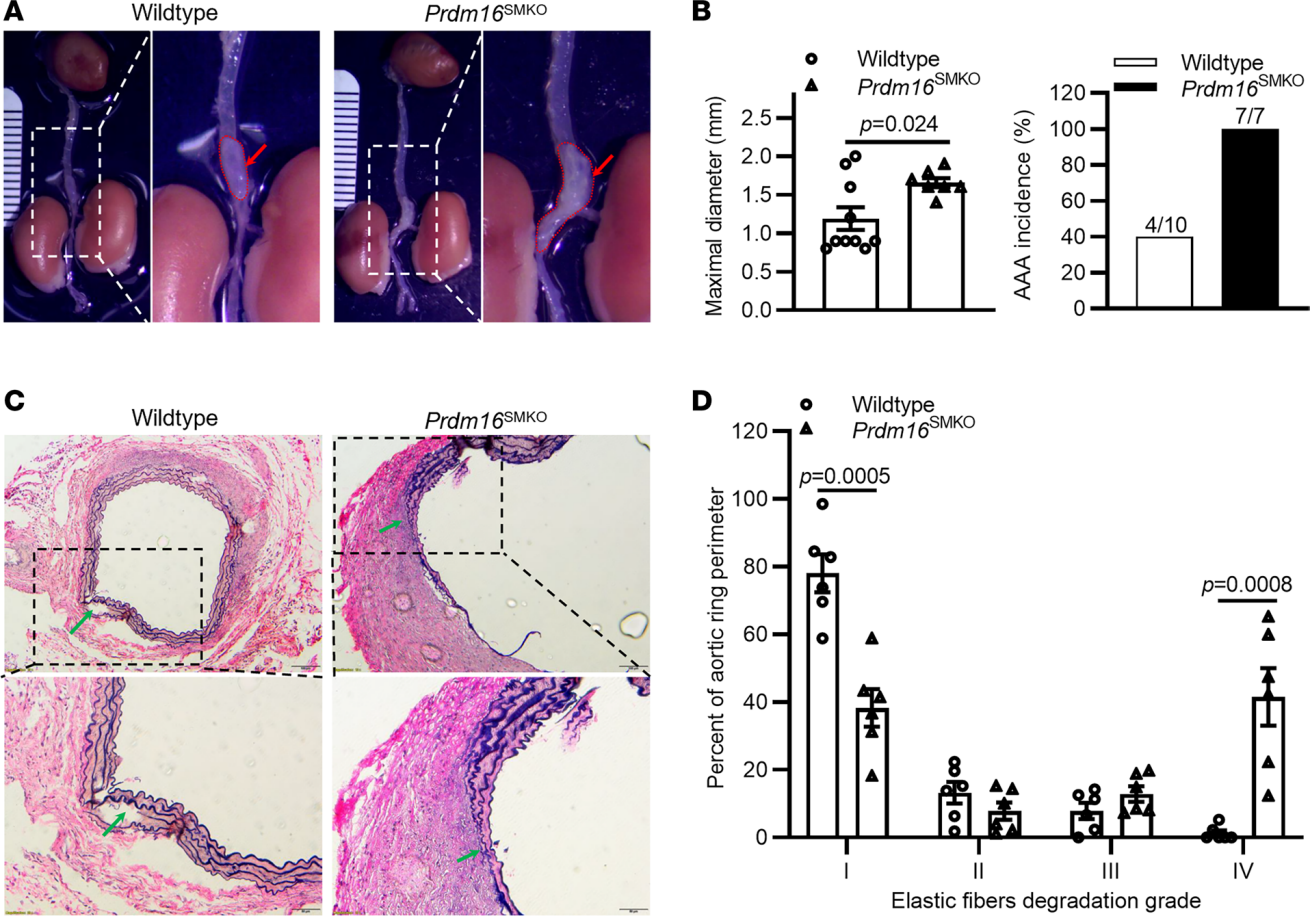
VSMC-specific *Prdm16* knockout aggravates elastase-induced abdominal aortic aneurysm (AAA) in mice. In the periadventitial elastase model, the suprarenal segment of the abdominal aorta was exposed to 100% porcine pancreatic elastase for 10 minutes, and AAA formation was evaluated 2 weeks later. (**A**) Representative views of AAA morphology; red arrows indicate AAA lesion area. (**B**) The maximal diameter of the abdominal aorta was measured and the AAA incidence was calculated. (**C**) Representative Verhoeff–Van Gieson staining of the abdominal aorta; green arrows indicate the fragmentation/degradation of elastic fibers. (**D**) Quantification of the degree of elastic fiber degradation levels in the abdominal aortic wall. *n* = 10 control mice, *n* = 7 *Prdm16*^SMKO^ mice. Scale bars: 100 μm (**C**, upper) and 50 μm (**C**, lower). Data are presented as mean ± SEM. *P* values were calculated using 2-tailed Student’s *t* test (**B**) or 2-way ANOVA with Holm-Šidák multiple-comparison test (**D**).

To investigate the role of PRDM16 in AAA pathology in vivo, we next used the periadventitial elastase model (22) for AAA induction in *Prdm16*^SMKO^ and wild-type control mice. We found that *Prdm16*^SMKO^ mice exhibited more severe AAA lesions compared with control mice (Figure 3A). Furthermore, both the maximal diameter of the abdominal aorta and the AAA incidence in *Prdm16*^SMKO^ mice (1.66 ± 0.06 mm; 100% incidence) were significantly greater than those in wild-type control mice (1.19 ± 0.15 mm; 40% incidence) (Figure 3B). We next performed Verhoeff–Van Gieson staining of the abdominal aorta and found that the fragmentation and degradation of elastin fibers in *Prdm16*^SMKO^ mice were significantly greater than that in wild-type control mice (Figure 3C). We also evaluated the degree of degradation of elastic fibers using the strategy shown in Supplemental Figure 4. We found that *Prdm16*^SMKO^ mice showed significantly decreased percentages of well-organized elastic fibers, but markedly increased percentages of greatly degraded elastic fibers (Figure 3D). These results indicate that PRDM16 deficiency in VSMCs aggravates elastase-induced AAA in mice.

### Prdm16 knockdown exacerbates inflammation and apoptosis in VSMCs.

**Figure 4:**
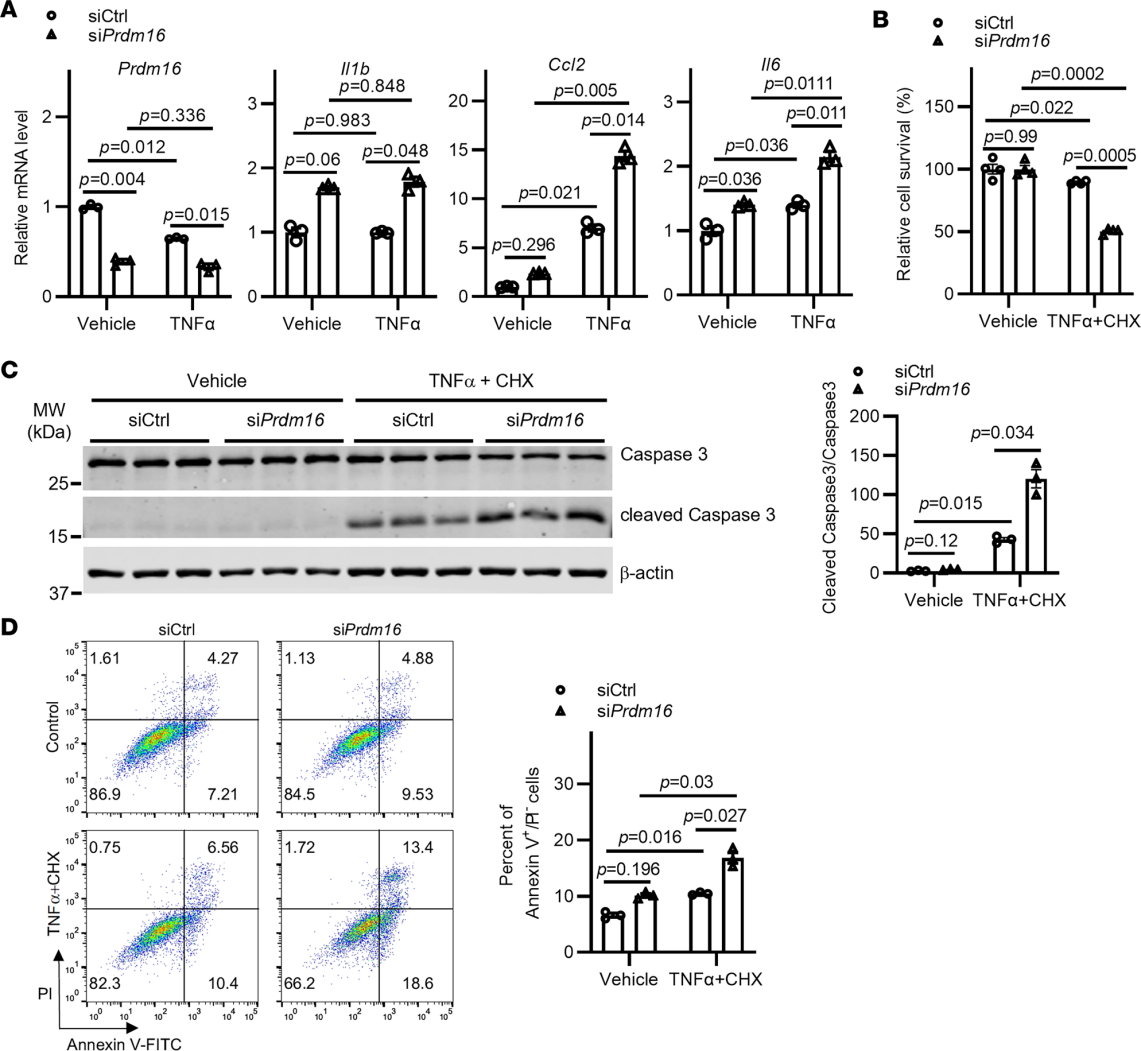
*Prdm16* knockdown exacerbates apoptosis in VSMCs. (**A**) A7r5 cells were transfected with siRNA-control (siCtrl, 10 nM) or siRNA-*Prdm16* (si*Prdm16*, 10 nM) for 48 hours. The cells were then treated with TNF-α (5 ng/mL) for 6 hours. Expression of proinflammatory genes was determined by qPCR (the siCtrl + Vehicle group, set as 1 for each gene, serves as control). (**B**–**D**) A7r5 cells were transfected with siCtrl (10 nM) or si*Prdm16* (10 nM) for 48 hours. The cells were then treated with TNF-α (10 ng/mL) plus cycloheximide (CHX, 20 mM) for 6 hours. (**B**) Cell survival was evaluated by MTT assay. (**C**) Apoptosis of A7r5 cells was assessed by the cleavage of caspase 3 using Western blotting. The level of cleaved caspase 3 relative to full-length caspase 3 is shown. (**D**) Apoptosis of A7r5 cells was determined by annexin V/PI staining followed by flow cytometry analysis. The histogram shows the percentage of annexin V^+^/PI^–^ cells. Data are presented as mean ± SEM. *P* values were calculated using 2-way ANOVA with Holm-Šidák multiple-comparison test (**A**–**D**).

From the GO analysis, we found that the upregulated genes were significantly enriched in positive regulation of inflammation and apoptotic process, while the downregulated genes were significantly enriched in negative regulation of cell death and apoptotic process (Supplemental Table 2). Since inflammation and apoptosis of VSMCs are the major hallmarks of AAA pathology (23), we next determined whether PRDM16 is involved in inflammation and apoptosis of VSMCs. Consistent with the increased expression of proinflammatory genes in the abdominal aorta of *Prdm16*^SMKO^ mice, we found that *Prdm16* knockdown markedly increased *Il1b*, *Ccl2*, and *Il6* expression in A7r5 cells under TNF-α–stimulated conditions (Figure 4A). Moreover, similar changes in *Il1b* and *Il6* expression upon *Prdm16* knockdown were found in primary VSMCs, except that *Ccl2* showed comparable expression upon *Prdm16* knockdown under basal conditions (Supplemental Figure 5A). To induce apoptosis in A7r5 cells, we used TNF-α and cycloheximide (CHX), a widely used approach, since it has been shown that a combination of TNF-α and CHX could more efficiently reduce VSMC viability than TNF-α or CHX alone (24), and the suppression of the synthesis of short-lived antiapoptotic proteins by CHX is required for TNF-α–induced apoptosis (25). When treated with TNF-α and CHX, VSMCs showed a lower survival rate upon *Prdm16* knockdown (Figure 4B). Moreover, *Prdm16* knockdown markedly promoted the cleavage of caspase 3 (the primary effector caspase involved in apoptosis) (Figure 4C). Consistently, annexin V/propidium iodide (PI) staining assays revealed that the percentage of apoptotic cells (annexin V^+^/PI^–^) was significantly elevated upon *Prdm16* knockdown (Figure 4D). These data indicate that *Prdm16* knockdown exacerbates apoptosis in VSMCs.

### ADAM12 is a target gene of PRDM16-dependent repression.

**Figure 5:**
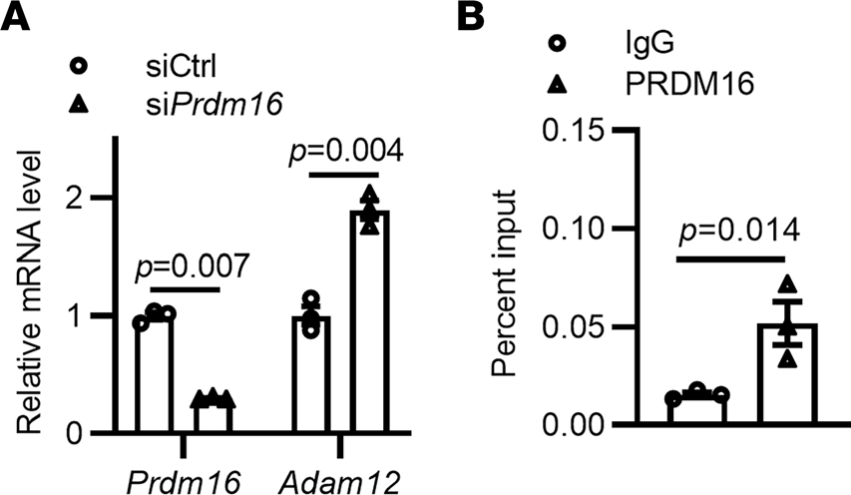
*Adam12* is a transcriptional target of PRDM16. (**A**) Primary rat VSMCs were transfected with control siRNA (siCtrl; 10 nM) or si*Prdm16* (10 nM) for 48 hours, and the relative gene expression levels were determined by qPCR. (**B**) Primary VSMCs were cultured, and ChIP assay followed by qPCR analysis was performed to determine the binding of PRDM16 to the promoter region of *Adam12*. Data are presented as mean ± SEM (*n* = 3). *P* values were calculated by 2-tailed Student’s *t* test.

Based on the RNA-seq data of the abdominal aorta, we found that the expression of ECM-related genes (including *Eln*, *Lox*, *Col3a1*, and *Ccn1*) was significantly reduced in *Prdm16*^SMKO^ mice (Figure 1E). It has been shown that several proteinase families, including matrix metalloproteinases (MMPs, the most extensively studied ECM-degrading proteinases), ADAMs, ADAM with thrombospondin motifs (ADAM-TSs), and serine/cysteine proteinases (including cathepsins and granzymes), are involved in ECM remodeling (4, 5). We found that *Mmp2* expression was slightly but significantly increased upon *Prdm16* knockdown in VSMCs and A7r5 cells, while *Mmp9* expression was significantly decreased upon *Prdm16* knockdown under TNF-α–stimulated conditions (Supplemental Figure 5, B and C). Moreover, the enzymatic activities of MMP2 and MMP9 showed no appreciable changes (Supplemental Figure 5, D and E), suggesting that MMPs may not contribute significantly to ECM remodeling in the *Prdm16*^SMKO^ mice. Next, we conducted a search for overlapping genes between the ECM-remodeling proteinases in RNA-seq data of the abdominal aorta and potential PRDM16 target genes in the data set of CHEA Transcription Factor Targets in Harmonizome (26). We focused our attention on a specific ADAM, namely ADAM12 (as shown in Supplemental Figure 6). We found that *Adam12* expression was significantly increased in VSMCs upon *Prdm16* knockdown (Figure 5A), while other ADAM family members that are highly expressed in VSMCs (including *Adam1a*, *Adam4*, *Adam9*, *Adam10*, *Adam15*, *Adam17*, *Adam19*, *Adam22*, and *Adam33*) showed PRDM16-independent expression (Supplemental Figure 7). By using chromatin immunoprecipitation (ChIP) assays, we confirmed that exogenously overexpressed PRDM16 could bind to the promoter region of *Adam12* in both primary VSMCs (Supplemental Figure 8A) and A7r5 cells (Supplemental Figure 8B). We further verified that the endogenous PRDM16 also efficiently binds to the promoter region of *Adam12* in primary VSMCs (Figure 5B). Thus, these data indicate that PRDM16 transcriptionally represses expression of *Adam12* in VSMCs.

### Reverse association between the expression of Prdm16 and Adam12 in both VSMCs and aortas.

**Figure 6:**
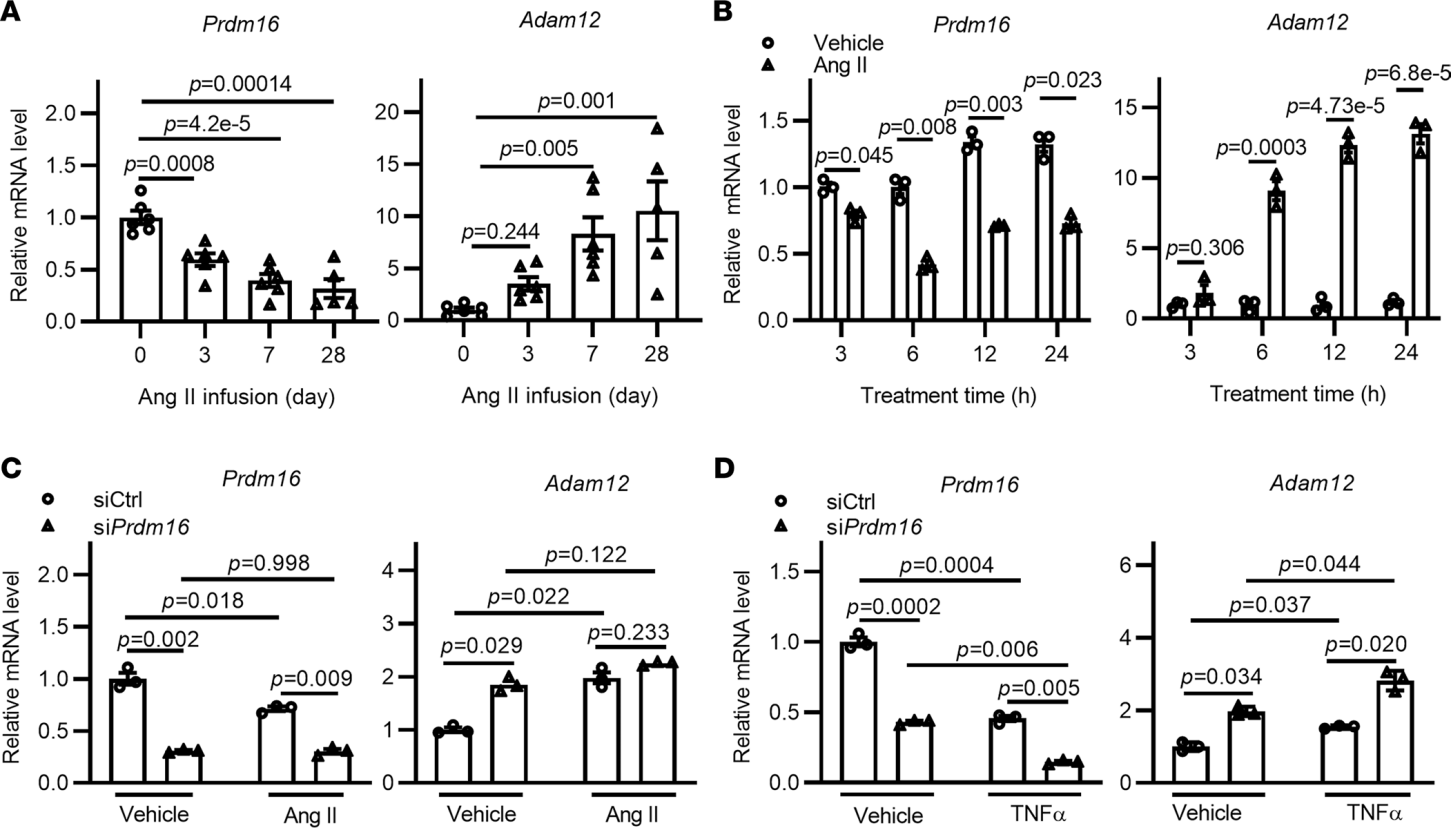
Reverse association between the expression of *Prdm16* and *Adam12* in VSMCs and aortas. (**A**) Relative *Prdm16* and *Adam12* mRNA expression in the abdominal aorta of C57BL/6J mice infused with Ang II (1000 ng/kg/min) for the indicated time was determined by qPCR (*n* = 5–6 mice for each group, day 0 group was set as 1, serves as control). (**B**) Primary rat VSMCs were treated with vehicle or Ang II (1 mM) for the indicated time, and the relative *Prdm16* and *Adam12* mRNA levels were analyzed by qPCR (cells treated with vehicle for 3 hours serve as control and is set as 1). (**C** and **D**) Primary rat VSMCs were transfected with control siRNA (siCtrl; 10 nM) or si*Prdm16* (10 nM) for 48 hours. The cells were then treated with Ang II (1 mM) (**C**) or TNF-α (5 ng/mL) (**D**) for 4 hours. Expression of *Prdm16* and *Adam12* was determined by qPCR (the siCtrl + Vehicle group, set as 1 for each gene, serves as control). *n* = 3, data are presented as mean ± SEM. *P* values were calculated using 1-way ANOVA with Holm-Šidák multiple-comparison test (**A**) or 2-way ANOVA with Holm-Šidák multiple-comparison test (**B**–**D**).

To determine the expression changes of *Prdm16* and *Adam12* in aortas, we treated VSMCs or wild-type mice with angiotensin II (Ang II), a critical factor that promotes AAA development in mice. We found that the expression of *Prdm16* was significantly reduced, while the expression of *Adam12* was markedly induced in the aorta of C57BL/6J mice infused with Ang II for different time points (Figure 6A). Consistently, treatment of primary VSMCs with Ang II significantly decreased expression of *Prdm16*, while the expression of *Adam12* was increased 10- to 15-fold after treatment with Ang II for 12–24 hours (Figure 6B). Although Ang II repressed *Prdm16* expression and induced *Adam12* expression, Ang II did not augment the transcriptional repressive effect of PRDM16 on *Adam12* (Figure 6C). Next, we switched the stimulation of VSMCs to TNF-α, as VSMC inflammation and apoptosis are another critical arm in promoting AAA formation. As shown in Figure 6D, TNF-α treatment significantly repressed expression of *Prdm16*, while it significantly induced expression of *Adam12* in VSMCs, and *Prdm16* knockdown augmented *Adam12* expression upon TNF-α stimulation. We also determined the mRNA and protein expression of ADAM12 in human AAA specimens. As shown in Supplemental Figure 9, both the mRNA and protein expression of ADAM12 was markedly increased in human AAA specimens compared with control, indicating that the PRDM16/ADAM12 axis was also involved in human AAA formation. Taken together, these in vivo and in vitro studies provide evidence for an association between PRDM16 and ADAM12 during AAA development.

### Prdm16 knockdown increases VSMC apoptosis in an ADAM12-dependent fashion.

**Figure 7:**
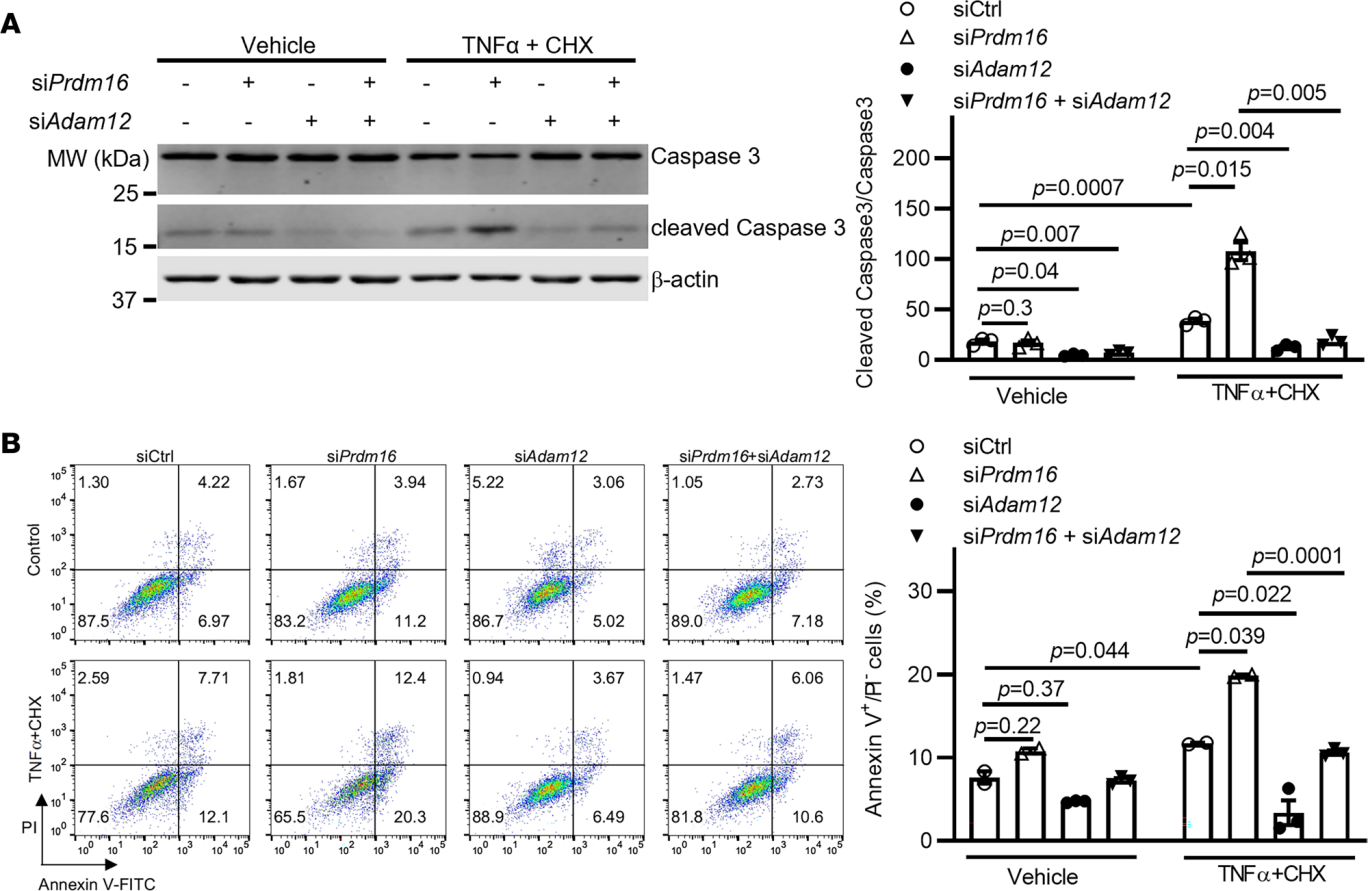
Knockdown of *Adam12* reverses apoptosis induced by PRDM16 deficiency. (**A** and **B**) A7r5 cells were transfected with the indicated siRNA (a total of 15 nM, 7.5 nM each of si*Prdm16* and si*Adam12*, with 7.5 nM siCtrl for the single gene knockdown to maintain constant siRNA concentration) for 48 hours. The cells were then treated with TNF-α (10 ng/mL) plus cycloheximide (CHX, 20 mM) for 6 hours. (**A**) Apoptosis of A7r5 cells was assessed by the cleavage of caspase 3 using Western blotting. The level of cleaved caspase 3 relative to full-length caspase 3 is shown. (**B**) Annexin V/PI staining followed by flow cytometry analysis; the histogram shows the percentage of annexin V^+^/PI^–^ cells. Data are presented as mean ± SEM. *P* values were calculated using 2-way ANOVA with Holm-Šidák multiple-comparison test (**A** and **B**).

To investigate the possible involvement of ADAM12 in apoptosis induced by PRDM16 deficiency in VSMCs, we next performed *Adam12* and *Prdm16* double knockdown experiments. As shown in Figure 7A, *Adam12* knockdown efficiently inhibited the cleavage of caspase 3 and the proapoptotic effects of *Prdm16* deficiency in VSMCs. Consistently, the annexin V/PI staining assay showed that the percentage of VSMCs that became apoptotic (annexin V^+^/PI^–^) upon *Adam12* and *Prdm16* double knockdown was significantly lower than that in *Prdm16*-deficient VSMCs (Figure 7B). In summary, these findings establish that *Adam12* is required for apoptosis associated with *Prdm16* deficiency in VSMCs.

## Discussion

AAA is the most common type of aortic aneurysm, with a high risk of rupture and dissection, and it is typically asymptomatic until complications occur (27). Currently, effective pharmacological treatment for AAA is still lacking, partly due to a limited understanding of the underlying mechanisms for the development and progression of AAA. As a transcriptional regulator, PRDM16 has been recently shown to play critical roles in cardiac development and function (12–14). Cardiac-specific depletion of PRDM16 results in cardiac hypertrophy (13, 14) and left ventricular noncompaction cardiomyopathy (12). Apart from the heart, *Prdm16* has also been shown to be highly expressed in the aorta (16). According to the GTExPortal, the transcripts per million value of *Prdm16* in the aorta is the highest among all displayed human tissues. However, the functions of PRDM16 in the aorta are unknown. In this study, we investigated the functions of PRDM16 in VSMCs.

By RNA-seq analysis, we found that *Prdm16*^SMKO^ mice already showed extensive changes in the expression of genes associated with ECM remodeling and inflammation in the abdominal aorta under normal housing conditions without any pathological stimuli. The expression of *Ccn3* (encoding cellular communication network factor 3), which has been identified as a negative regulator of AAA formation by ameliorating inflammatory cell infiltration (28), was markedly downregulated in the abdominal aorta of *Prdm16*^SMKO^ mice. We also found that the antigen processing and presentation–associated genes (including *H2-Aa*, *H2-D1*, and *H2-Q4*), *Thbs4* (thrombospondin-4), and *Ccl11* (eotaxin) were upregulated in the abdominal aorta of *Prdm16*^SMKO^ mice. H2-D1 has been shown to be upregulated in aortas with atherogenic lesions (29). As an ECM protein, THBS4 has been shown to promote vascular inflammation and was positively associated with atherogenesis (30). THBS4 deficiency increased ECM deposition in the heart of mice and contributes to cardiac hypertrophy (31). THBS4 conventional knockout mice treated with Ang II showed higher incidence of aneurysm compared with control mice (32). Moreover, Ang II–treated THBS4-deficient aortas displayed pronounced inflammation and aortic dissections in the outer medial layer of the arteries (33). CCL11 has been shown to be a potent eosinophil chemoattractant; it is abundantly present in atherosclerotic plaques and induces SMC migration (34). Inactivation of *Lox* (lysyl oxidase) has been shown to result in aortic aneurysms (35–37). We confirmed that *Lox* expression was significantly downregulated in the abdominal aorta of *Prdm16*^SMKO^ mice. Therefore, the changes in the above-mentioned genes in the abdominal aorta of *Prdm16*^SMKO^ mice suggest that deficiency of *Prdm16* in VSMCs is associated with development of aortopathies. Indeed, we demonstrated that deficiency of *Prdm16* in VSMCs accelerated AAA development induced by perivascular application of elastase. Although we did not investigate in detail the potential contributions of the genes mentioned above to the increased AAA formation in *Prdm16*^SMKO^ mice, these genes will undoubtedly cooperate with each other in facilitating ECM remodeling and inflammation in the abdominal aorta of *Prdm16*^SMKO^ mice. We acknowledge that a limitation of our study is that we focused on *Prdm16*^SMKO^ effects on VSMCs. Previous research demonstrated that PRDM16 is highly expressed in small intestinal crypts and is involved in regulating small intestinal epithelial renewal by controlling region-specific metabolism (38). *Myh11*-CreER^T2^ mice have also been shown to exhibit Cre activity in SMCs in the gastrointestinal system (39). Thus, PRDM16 may affect cholesterol absorption/metabolism in the gastrointestinal SMCs, which will be addressed in future studies using other models.

During the development of AAA, VSMCs undergo apoptosis because of both intrinsic and environmental changes, including ECM remodeling (4). For the first time to our knowledge, we documented that PRDM16 transcriptionally repressed *Adam12* expression. ADAM12 can degrade various ECM proteins (40). ADAM12 is a member of the disintegrin and metalloproteinase family (ADAM), whose roles in aortic aneurysm are less explored. In contrast, the most studied member, ADAM17, has been found to play critical roles in aortic aneurysm development in both mice and humans (41). ADAM12 was initially identified as a regulator of heart function because chemical antagonism of ADAM12 provides potent inhibition of cardiac hypertrophy (42). More recently, ADAM12 was identified as a diagnostic indicator to classify ruptured intracranial aneurysm (IA) from unruptured IA along with several other biomarkers (43), suggesting a potential role in aortic aneurysm. In our study, we found that ADAM12 is a highly expressed ADAM in VSMCs and a transcriptional target of PRDM16, with its expression inhibited by PRDM16. This is in agreement with a recent study reporting that ADAM12 expression was increased upon PRDM16 depletion in the heart of mice (12). In our study, we found that ablation of PRDM16 promotes apoptosis of VSMCs, which might be associated with enhanced ADAM12 expression in VSMCs. The independent stimulations and functional assays provide robust evidence that both PRDM16 and ADAM12 are involved in AAA development. Future studies will further investigate the role of ADAM12 in VSMCs and other cell types like macrophages and fibroblasts in the abdominal aorta. Additionally, it has been demonstrated that PRDM16 could functionally interact with peroxisome proliferator-activated receptor γ (PPARγ) (44). Although currently there are no available agonists for PRDM16, it has been shown that PPARγ agonists could potently increase the half-life of the PRDM16 protein (45) and PPARγ agonists attenuate inflammation in aortic aneurysm patients (46). A recent study identified CUL2-APPBP2 as the ubiquitin E3 ligase that determines PRDM16 protein stability by catalyzing its polyubiquitination (47). Future studies should further develop specific compounds targeting PRDM16 and investigate their therapeutic potential in AAA. Moreover, it is possible that targets of PRDM16 could play clinically relevant roles and serve as targets for diagnosis or intervention. In that regard, it is remarkable that ADAM12 was recently proposed as a diagnostic classifier for rupture of intracranial aneurysms (43). As AAA cohorts increase in number and stratification by risk factors becomes feasible, it is possible that the PRDM16/ADAM12 axis may be confirmed as a key factor in human AAA.

In summary, the deficiency of PRDM16 in VSMCs leads to overall changes in the aorta, including increased VSMC apoptosis, ECM remodeling, and inflammation, which contribute to the development of AAA. This conclusion raises the possibility that elevation of PRDM16 protein levels or transcriptional activity or inhibition of its target, ADAM12, would protect from AAA development and progression.

## Methods

### AAA model.

*Prdm16^fl/fl^* mice (stock number 024992) and *Myh11*-CreER^T2^ mice (stock number 019079) were from the Jackson Laboratory and were backcrossed to the C57BL/6J background. *Prdm16^fl/fl^* mice were crossbred with *Myh11*-CreER^T2^ mice (carrying the CreER cassette on the Y chromosome) to generate *Prdm16*^SMKO^ mice. Eight- to 10-week-old male mice were treated with tamoxifen (75 mg/kg/day) in corn oil for 5 consecutive days by gavage. Two weeks later, the mice were used for AAA induction by application of 100% of porcine pancreatic elastase (PPE; Sigma-Aldrich) to the adventitial surface of the suprarenal abdominal aorta. The AAA incidence rate of this model is dependent on the incubation time with PPE. We optimized the PPE incubation time and found that incubation of aortas with PPE for 10 minutes was the optimal time window in our study. Briefly, the upper abdomen of mice was opened, and the suprarenal abdominal aorta was isolated and exposed to PPE for 10 minutes, followed by washing with 0.9% sodium chloride solution 3 times, wound closure, and recovery. After 2 weeks, mice were euthanized, and AAA formation was evaluated. Verhoeff–Van Gieson staining of the abdominal aorta was performed using the Elastic Stain Kit (ab150667, Abcam). All mice were maintained under normal housing conditions (12-hour light/dark cycle, 23°C) with free access to regular chow diet and water.

### Cell procedures.

Primary VSMCs were isolated from the suprarenal segment of the aorta of 10-week-old male rats as previously described (48) and cultured in DMEM/F12 (1:1; Gibco) supplemented with 10% fetal bovine serum (FBS; Gibco). Primary VSMCs of passages 3 to 10 were used for the experiments. The A7r5 cell line was obtained from ATCC (CRL-1444) and was maintained in DMEM supplemented with 10% FBS. Prior to experiments, primary VSMCs and A7r5 cells were serum starved in Opti-MEM I (Gibco) supplemented with 0% and 0.5% FBS, respectively. All cells were confirmed negative for mycoplasma. Recombinant rat TNF-α was obtained from R&D Systems. PRDM16 siRNA (target sequence: UGACAGUUUAGCCGGGAAA) and ADAM12 siRNA (target sequence: CGGGAUGAAUCACGACACA) were obtained from Dharmacon, Horizon.

### Western blot analysis.

Whole-cell lysates from VSMCs were prepared with RIPA buffer supplemented with protease inhibitor cocktail (Roche), and were separated using SDS-PAGE, followed by transfer onto nitrocellulose membranes. The membranes were blocked in 5% nonfat dry milk and probed with the following primary antibodies: anti–caspase 3 (1:1000; catalog 9662, Cell Signaling Technology), anti–β-actin (1:2000; catalog 3700, Cell Signaling Technology), and anti-ADAM12 (1:1000; catalog A7940, ABclonal). The signals were captured and quantified using Image Studio (version 3.1, Odyssey CLx).

### qPCR.

Total RNA from VSMCs and aortas were isolated with the RNeasy Mini kit (catalog 74106, Qiagen) and RNeasy Micro kit (catalog 74004, Qiagen), respectively. cDNA samples were synthesized by using the SuperScript III First-Strand Synthesis System (catalog 18080, Life Technologies). The relative mRNA expression was calculated using the 2^–ΔΔCt^ method, and *Gapdh* was used as the housekeeping gene. All the primers used in this study are listed in Supplemental Table 3.

### Flow cytometry analysis of apoptosis.

A7r5 cells were transfected with 10 nM siRNA for 48 hours, and then treated with 10 ng/mL TNF-α and 20 μM CHX for 6 hours. The cells were then trypsinized and stained with FITC Annexin V/PI (BD Biosciences) according to the manufacturer’s instructions, followed by fluorescence-activated cell sorting (FACS) analysis using a MoFlo Astrios Cell Sorter (Beckman Coulter) at the flow cytometry core of the University of Michigan. The results were analyzed using FlowJo software (version 10.8.1, BD Biosciences).

### ChIP assay.

ChIP assays were performed using the SimpleChIP Enzymatic Chromatin IP Kit (catalog 9003, Cell Signaling Technology) according to the manufacturer’s instructions. Briefly, VSMCs were crosslinked in 1% paraformaldehyde for 10 minutes at room temperature. The digested chromatin was immunoprecipitated with an anti-FLAG antibody (1 μg; catalog 14793, Cell Signaling Technology), anti-PRDM16 (2 μg; catalog ab106410, Abcam), or equal amount of normal IgG (catalog 2729, Cell Signaling Technology) overnight at 4°C with rotation. The beads were extensively washed with low- and high-salt buffers and the eluted DNA was analyzed by qPCR. The primers used in this assay are listed in Supplemental Table 3.

### Immunofluorescent staining.

Immunofluorescent staining was conducted as previously described (49, 50). Briefly, human aortic samples were fixed in 10% formalin for 24 hours and embedded in paraffin at the In Vivo Animal Core of the University of Michigan. The serial sections (5 μm thick, 200 μm apart) were deparaffinized, rehydrated, and boiled in citrate buffer (catalog 00-5000, Invitrogen) for epitope retrieval. The sections were incubated with primary antibodies against PRDM16 (1:100; catalog ab106410, Abcam) and SM22α (1:100; catalog ab10135, Abcam) at 4°C overnight. Slides were mounted with ProLong Gold Antifade Mountant with DAPI (catalog P36935, Invitrogen) and images were collected with an Olympus DP73 microscope.

### RNA-seq.

RNA samples from the abdominal aorta of mice with RNA integrity number (RIN) greater than 7 were submitted to the Advanced Genomics Core of the University of Michigan for RNA-seq, as we previously described (51).

### Statistics.

Statistical analyses were performed using GraphPad Prism software (version 9.3.1) or R (version 4.1.1, for RNA-seq analysis). All data were tested for variance and normality. For comparisons between 2 groups, Student’s *t* test was used. For comparisons among 3 or more groups, 1-way or 2-way ANOVA followed by Holm-Šidák multiple-comparison test was used. Differences with *P* less than 0.05 were considered statistically significant. Data are presented as mean ± SEM.

### Study approval.

The animal study protocols were approved by the Institutional Animal Care and Use Committee of the University of Michigan.

### Data availability.

The RNA-seq data are available in the NCBI Gene Expression Omnibus (GEO) with accession number GSE222440.

## Author contributions

LC and ZW designed the research. ZW, XZ, GZ, YG, WM, J Zhong, and LC performed experiments. ZW, HL, J Zhang, and LC analyzed data. LC, ZW, and YEC provided critical ideas. ZW, LC, and MGB wrote and revised the manuscript.

## Supplementary Material

Supplemental data

Supplemental data set 1

## Version 1 04/20/2023

In-Press Preview

## Version 2 06/08/2023

Electronic publication
