# Supplementary material for: PRDM16 deficiency in vascular smooth muscle cells aggravates abdominal aortic aneurysm
Source: JCI Insight. 2023 Jun 8;8(11):e167041. doi: 10.1172/jci.insight.167041 (PMC10393233; doi:10.1172/jci.insight.167041)
Supplement: Supplemental data [file jciinsight-8-167041-s063.pdf]

SUPPLEMENTARY INFORMATION

PRDM16 deficiency in vascular smooth muscle cells aggravates abdominal aortic aneurysm

Zhenguo Wang, Xiangjie Zhao, Guizhen Zhao, Yanhong Guo, Haocheng Lu, Wenjuan Mu, Juan Zhong, Minerva Garcia-Barrio, Jifeng Zhang, Y. Eugene Chen, and Lin Chang

Table of Contents:

Supplementary Figure S1
Supplementary Figure S2
Supplementary Figure S3
Supplementary Figure S4
Supplementary Figure S5
Supplementary Figure S6
Supplementary Figure S7
Supplementary Figure S8
Supplementary Figure S9
Supplementary Table 1
Supplementary Table 2
Supplementary Table 3
Supplementary Table 4

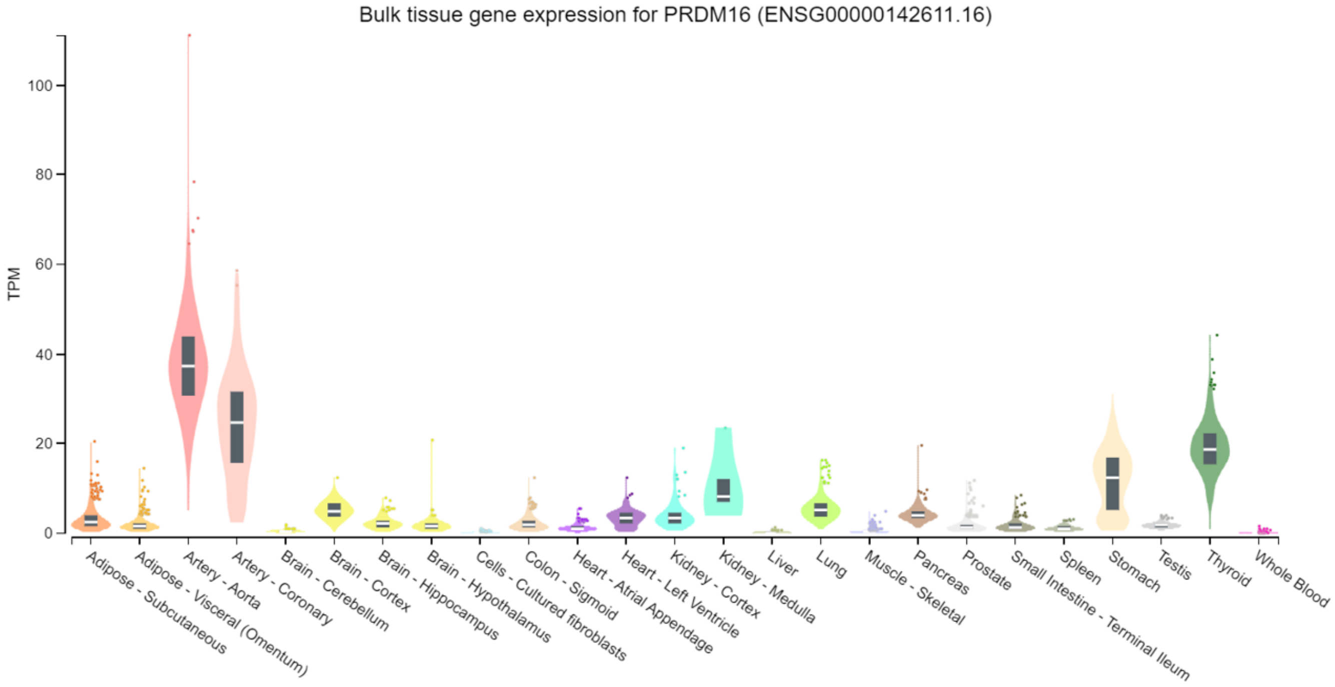

Figure S1. PRDM16 mRNA levels in different tissues.
The Genotype-Tissue Expression (GTEx) Project was supported by the Common Fund of the Office of the Director of the National Institutes of Health, and by NCI, NHGRI, NHLBI, NIDA, NIMH, and NINDS. The data used for the analyses described in this manuscript were obtained from: GTEx Portal and dbGaP Accession phs000424.v8.p2 and last updated on 9/8/2022.

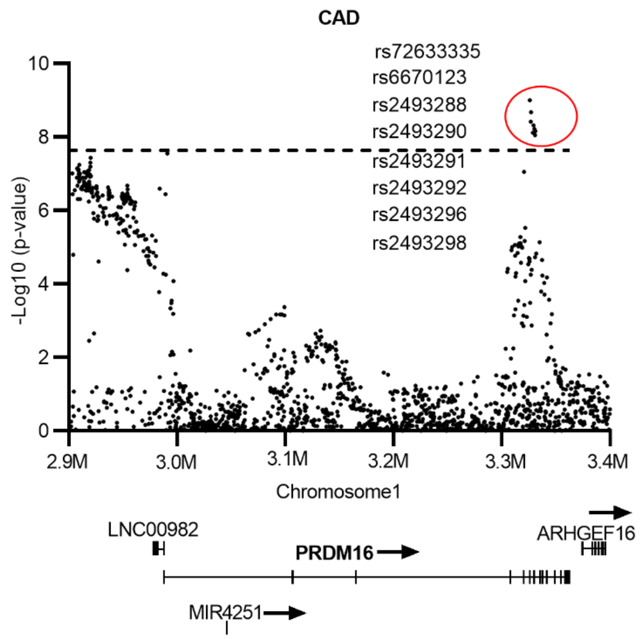

Figure S2. Association between SNPs in PRDM16 and the risk of coronary artery disease (CAD) and hypertension in population. The GWAS data of CAD were downloaded from a genome-wide association study conducted by Van der Harst P et al (PMID: 29212778), which included 34,541 CAD cases and 261,984 controls from the UK Biobank resource, followed by replication in 88,192 cases and 162,544 controls from CARDIoGRAMplusC4D. The data of Hypertension were downloaded from Oriol Canela-Xandri et al (PMID: 30349118), which conducted an atlas of genetic associations for 118 non-binary and 660 binary traits of 452,264 UK Biobank participants of European ancestry, including 120,333 hypertension cases and 331,931 controls. The FINEMAP shows the significant associations of single nucleotide polymorphisms (SNPs) including rs72633335, rs6670123, rs2493288, rs2493290, rs2493291, rs2493292, rs2493296, and rs2493298 (the dots in red circle) are associated with a higher risk of developing of CAD and hypertension.

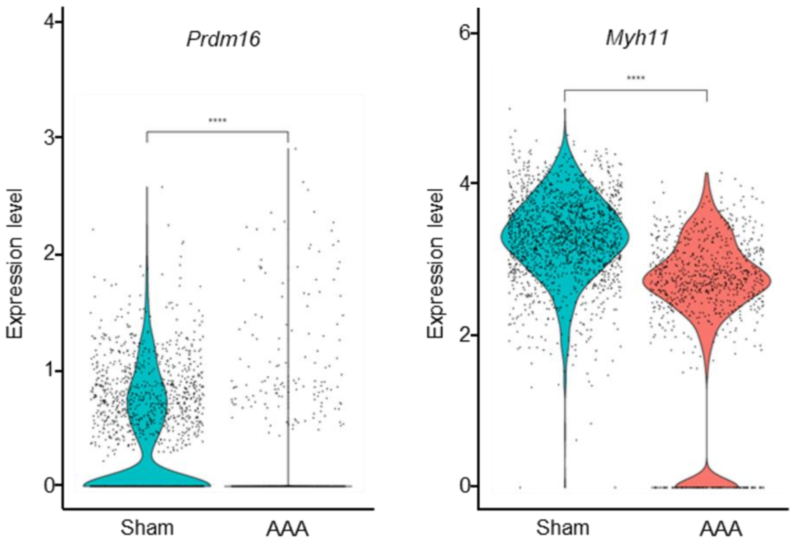

Figure S3. *Prdm16* mRNA expression in AAA lesions in mice.
The mRNA expression levels of *Prdm16* and *Myh11* in VSMCs from three single-cell RNA-seq datasets (GSE152583, GSE164678, PRJCA006049) were shown in Violin plots. Wilcoxon Rank-sum test (Mann-whitney U test) was used to calculate the statistical significance, ****p<0.0001.

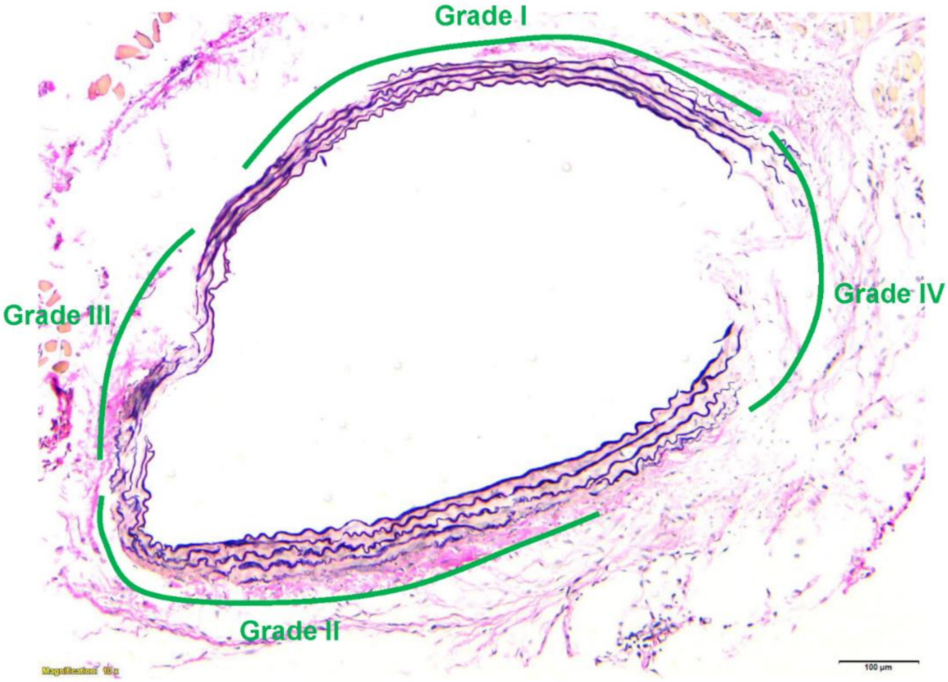

Figure S4. Definition of the degradation levels of elastic fibers. To evaluate the extent of degradation of elastic fibers, we defined a strategy to calculate the percentage of the length of different degradation levels of elastic fibers to the whole outside perimeter of the aorta. Grade I indicates that the elastin fibers are intact and well-organized; Grade II indicates that some of the elastin fibers are partially degraded, discontinuous, while the remaining elastic fibers are intact and well-organized; Grade III indicates that most of the elastic fibers are degraded and discontinuous; Grade IV indicates that almost all the elastic fibers are degraded. Scale bar, 100 µm.

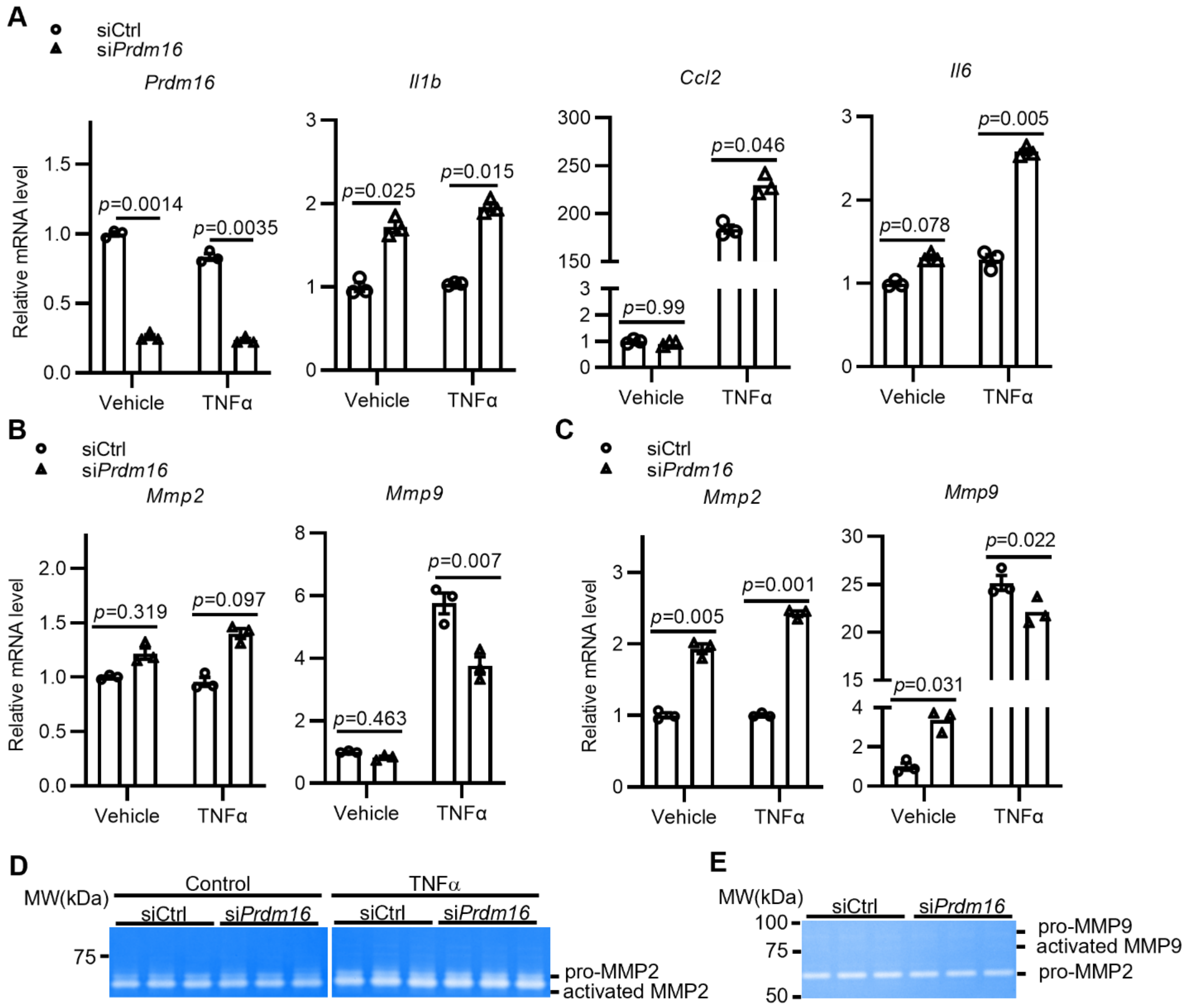

Figure S5. Expression of proinflammatory genes and MMP zymography analysis. A, Primary VSMCs were transfected with the indicated siRNA for 48 hours. The cells were then treated with TNFα (5 ng/mL) for 6 hours. Expression of proinflammatory genes (*Il1b*, interleukin-1 beta; *Ccl2*, C-C motif chemokine 2; *Il6*, interleukin-6) were determined by qPCR (the siCtrl+Vehicle group serves as control). **B** and **C**, Expression of matrix metalloproteinase (Mmp) genes were determined by qPCR in primary rat VSMCs (**B**) and A7r5 cells (**C**). **D** and **E**, MMP zymography analysis were performed in conditional media from primary rat VSMCs (**D**) and A7r5 cells (**E**). Data are presented as mean±SEM, *p* values were calculated by two-way ANOVA with Holm-Sidak multiple comparisons test (**A**-**C**).

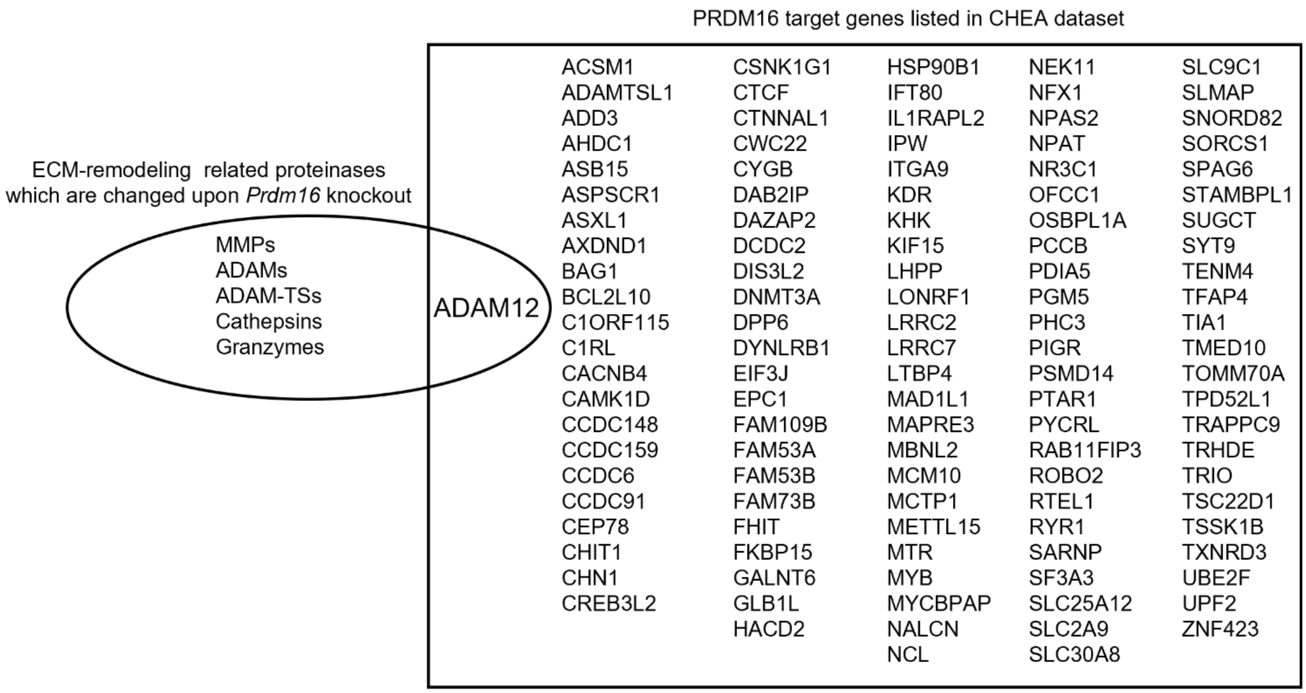

Figure S6. ADAM12 is an ECM-remodeling gene of PRDM16-dependent repression. Venn diagram shows the overlapping genes between ECM-remodeling related proteinases and PRDM16 target genes. PRDM16 target genes were obtained from the dataset of CHEA Transcription Factor Targets in Harmonizome.

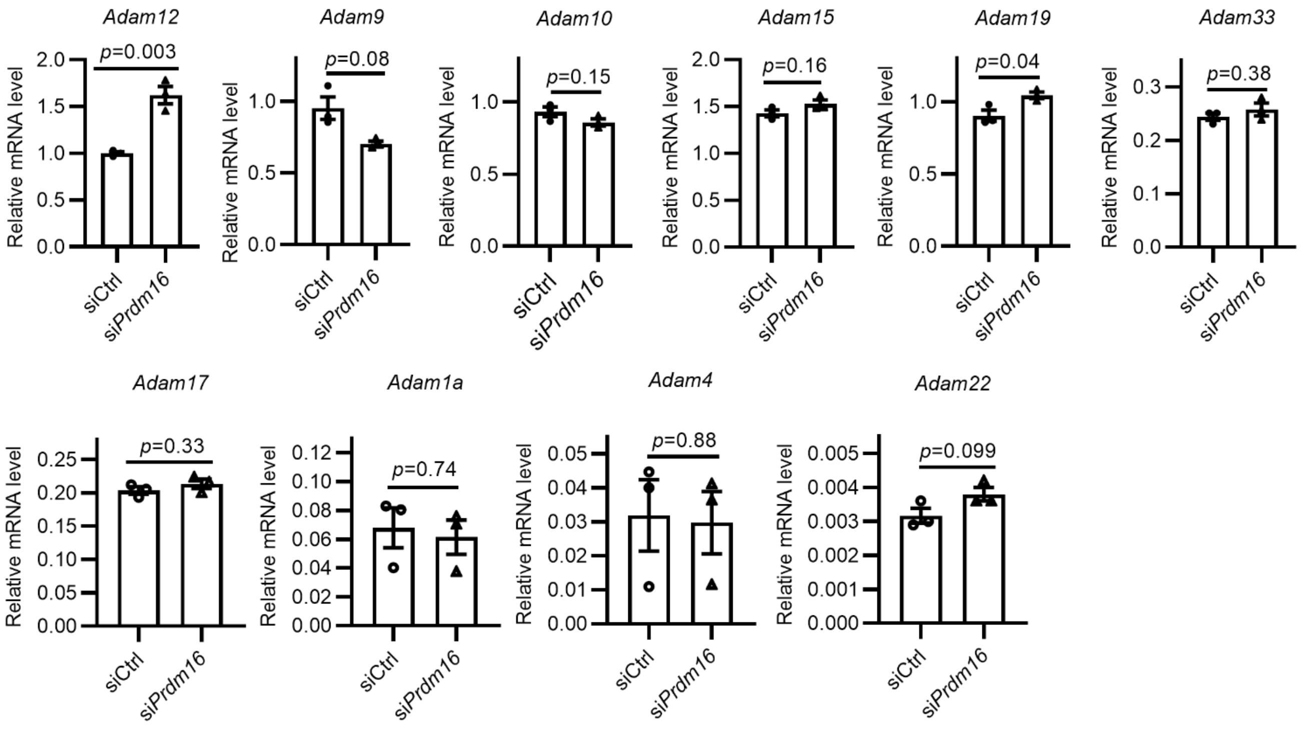

Figure S7. The expression of Adam family members in VSMCs upon *Prdm16* knockdown. Primary rat VSMCs were transfected with siCtrl (10 nM) or si*Prdm16* (10 nM) for 48 hours, and the relative gene expression levels were determined by qPCR. All data are normalized to the expression levels of *Adam12* in siCtrl VSMCs. Data are presented as mean±SEM, n=3. *p* values were calculated by multiple unpaired *t* tests with Holm-Sidak multiple comparisons correction.

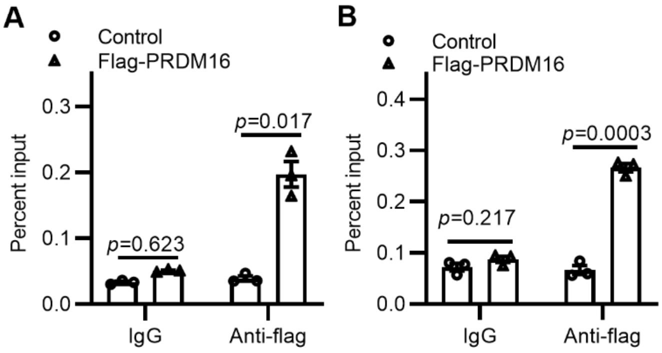

Figure S8. PRDM16 binds to the promoter region of *Adam12* in VSMCs. Primary VSMCs (**A**) or A7r5 cells (**B**) were infected with lentivirus carrying empty vector (control) or PRDM16 (Flag-PRDM16). ChIP assay followed by qPCR analysis was performed to determine the binding of PRDM16 to the promoter region of *Adam12*. Data are presented as mean±SEM (n=3), *p* values were calculated by two-way ANOVA with Holm-Sidak multiple comparisons test (A-B).

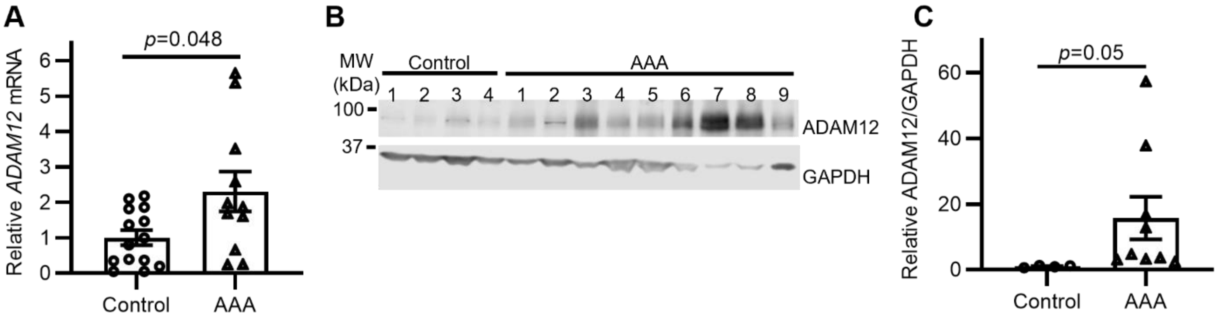

Figure S9. The mRNA and protein expression of ADAM12 were markedly increased in human AAA specimens. A, The mRNA expression of *ADAM12* were determined by qPCR. n=14 control, 11 AAA (Patients´ information are shown in Table S1). **B**, The protein expression of ADAM12 were determined by western blot. n=4 control, 9 AAA. Patients´ information are shown in Table S4. **C**, Quantification of the Western blot in (**B**). Data are presented as mean±SEM, *p* values were calculated by Student's *t* test (A and C).

Table S1. Patients´ information related to Figure 2

Sample #	Diagnosis	Age at time of surgery	Sex	Primary Race
1	Control: Dissection	61	M	Caucasian
2	Control: Dissection	55	M	Caucasian
3	Control: Dissection	64	M	Caucasian
4	Control: Dissection	65	M	Caucasian
5	Control: Aortic Stenosis	65	M	Caucasian
6	Control: Dissection	66	M	Caucasian
7	Control: Dissection	67	M	African American
8	Control: Dissection	67	M	Caucasian
9	Control: Dissection	68	M	African American
10	Control: Dissection	86	M	Caucasian
11	Control: Dissection	76	M	Caucasian
12	Control: Dissection	79	M	Caucasian
13	Control: Dissection	72	M	Caucasian
14	Abdominal aortic aneurysm	55	M	Caucasian
15	Abdominal aortic aneurysm	57	M	Caucasian
16	Abdominal aortic aneurysm	60	M	Caucasian
17	Abdominal aortic aneurysm	61	M	Caucasian
18	Abdominal aortic aneurysm	62	M	American Indian or Alaska Native
19	Abdominal aortic aneurysm	64	M	Caucasian
20	Abdominal aortic aneurysm	65	M	Caucasian
21	Abdominal aortic aneurysm	66	M	Caucasian
22	Abdominal aortic aneurysm	67	M	Caucasian
23	Abdominal aortic aneurysm	72	M	Caucasian
24	Abdominal aortic aneurysm	75	M	Caucasian
25	Abdominal aortic aneurysm	79	M	Caucasian
26	Abdominal aortic aneurysm	79	M	Caucasian
27	Abdominal aortic aneurysm	80	M	Caucasian
28	Abdominal aortic aneurysm	82	M	Caucasian

Table S2. Apoptosis-associated GO terms of DEGs

DEGs	GO term	Gene count	*p*-Value	Genes
Up	Positive regulation of apoptotic process	21	5.01E-04	*Bnip3l, Tgfb2, Ntrk3, Adam10, Bbc3, Ifit2, Olfm1, Sfrp4, Rassf2, Tfap4, Frzb, Eef1a2, Tnfsf10, Ankrd1, Emilin2, Txnip, Irf5, Pde5a, Sox4, Phlda3, Bok*
Down	Negative regulation of cell death	15	5.23E-06	*Ppp1r15a, Abcc1, Itgb3, Wnt16, Hspg2, Fgf2, Npas2, Chmp4c, Mfn2, Adnp2, Ccn3, Ccn1, Fermt2, Hspa1b, Hspa1a*
Down	Negative regulation of apoptotic process	29	0.035	*Btg2, Hspb6, Src, Hhip, Ihh, Ilk, Ptgs2, Higd1a, Fstl1, Gli2, Comp, Ccnd2, Mical1, Pdpn, Myo18a, Ccn1, Angpt4, Plk3, Jun, Egr3, Smad3, Siah2, Fn1, Id1, Pgr, Sgk1, Gas6, Hspa1b, Lims2*

Table S3. qPCR primers used in this study.

Genes	Forward primer	Reverse primer
Gapdh (mouse)	CTTTGTCAAGCTCATTTCCTGG	TCTTGCTCAGTGTCCTTGC
Prdm16 (mouse)	AGTCGGACAACCATGCACTT	GATCTCAGGCCGTTTGTCCA
Myh11 (mouse)	GACAACTCCTCTCGCTTTGG	GCTCTCCAAAAGCAGGTCAC
Acta2 (mouse)	CTGACAGAGGCACCACTGAA	CATCTCCAGAGTCCAGCACA
Eln (mouse)	AGTTCCTGGTGTTGGTCTTC	CCTTGGCTTTGACTCCTGTG
Fbn1 (mouse)	AATGAAGGCTATGAGGTGGC	TCTGTAGACTATACCCAGGCG
Fn1 (mouse)	CTTTGGCAGTGGTCATTTCAG	ATTCTCCCTTTCCATTCCCG
Lox (mouse)	CGATTTCCGCAAAGAGTGAAG	ATCAAGCAGGTCATAGTGGC
Col1a1 (mouse)	CATAAAGGGTCATCGTGGCT	TTGAGTCCGTCTTTGCCAG
Col3a1 (mouse)	GAAGTCTCTGAAGCTGATGGG	TTGCCTTGCGTGTTTGATATTC
Ccn1 (mouse)	GGAGGTGGAGTTAACGAGAAAC	GTGGTCTGAACGATGCATTTC
Ccn3 (mouse)	GACTCGTCTCTGCATCGTTC	GCTTTCAGGGATTTCTTGGTG
Il1b (mouse)	TCCTGTGTAATGAAAGACGGC	ACTCCACTTTGCTCTTGACTTC
Ccl2 (mouse)	GGTCTTCAGCACCTTTGAATG	ATTAAGGCATCACAGTCCGAG
Tnf (mouse)	CTTCTGTCTACTGAACTTCGGG	CAGGCTTGTCACTCGAATTTTG
Ccr2 (mouse)	GCTCTACATTCACTCCTTCCAC	ACCACTGTCTTTGAGGCTTG
Cd68 (mouse)	ACACTTCGGGCCATGTTTCT	GGGGCTGGTAGGTTGATTGT
Adgre1 (mouse)	ACCACAATACCTACATGCACC	AAGCAGGCGAGGAAAAGATAG
H2-Aa (mouse)	TCAAATTCCACCCCAGCTAC	CTATTTCTGAGCCATGTGATGTTG
ACTB (human)	GTCATTCCAAATATGAGATGCGT	GCTATCACCTCCCCTGTGTG
PRDM16 (human)	TTAAGGACATTGAGCCAGGTG	CAGCTTGGACTGGAAGAGTTC
ADAM12 (human)	CGAGAGTTTCAGAGGCAAGG	GAGCATTTGTCCATGTCATTCC
Gapdh (rat)	TCCAGTATGACTCTACCCACG	CACGACATACTCAGCACCAG
Prdm16 (rat)	AGAAGCACGAACACGAAGGT	ATCTCGCTGTTGGCGATGAA
Adam12 (rat)	TAGGTCAAGCTGCCCACACT	CCACAAACTCACTCCCAAGC
Mmp2 (rat)	GCTGATACTGACACTGGTACTG	CACTGTCCGCCAAATAAACC
Mmp9 (rat)	CTTGAAGTCTCAGAAGGTGGATC	CGCCAGAAGTATTTGTCATGG
Il1b (rat)	GCACAGTTCCCCAACTGGTA	TGTCCCGACCATTGCTGTTT
Ccl2 (rat)	CCAGAAACCAGCCAACTCTCA	CCAGAAGCGTGACAGAGACC
Il6 (rat)	AAGCCAGAGTCATTCAGAGC	GTCCTTAGCCACTCCTTCTG
Adam12_ChIP (rat)	AAATGAGCAGCCTGTCCGAG	CGTGGTAATGAGAACGGCGA
Adam1a (rat)	TGCTCTGGGGGTTTGTGTAA	TTTAGGTTAGCCGAGGTGGG
Adam4 (rat)	AAGGTATGCAGGCACACGAA	ATTGGCGGTCGCTACAGTTT
Adam8 (rat)	GCTCTAGGGTTAGTGTGCCC	GGCTACTGCTGGTGTCCATAA
Adam9 (rat)	CTGACCATCCCAACGTACAG	GCAGAGGTTCGATTCCAAAAC
Adam10 (rat)	TCCTGCCGTTTCACTCTGTC	CTGAATGTGCCCGAGTTCCT
Adam15 (rat)	GCCTTGTGCTAGTGGAGAGG	GGCTGTTTGGAGGCAAAGTG
Adam17 (rat)	GCCTAGCTCTTGAGTTTTCCCT	CCAGGACGAAAGGCACCAAA
Adam19 (rat)	CTGTGAGGAAAGTGGATGAGTC	TTGGTTGTCGCTGTTAGGG
Adam22 (rat)	TCAGGAAATTCGAGCCAGTG	GTCAGATGCAGTCACCTTTTG
Adam33 (rat)	CAACCTCTGGGCAGTGTTCA	ATTGGTGACTGGGCAATGGG

Table S4. Patients´ information related to Figure S9B-C

Sample ID	Diagnosis	Age at time of surgery	Sex	Primary Race
Control 1	Heart Transplant	54	M	Caucasian
Control 2	Heart Transplant	59	M	Caucasian
Control 3	Heart Transplant	65	M	Caucasian
Control 4	Heart Transplant	68	M	Caucasian
AAA 1	Aortic Disease, Abdominal Aortic Aneurysm	61	M	Caucasian
AAA 2	Aortic Disease, Abdominal Aortic Aneurysm	79	M	Caucasian
AAA 3	Aortic Disease, Abdominal Aortic Aneurysm	60	M	Caucasian
AAA 4	Aortic Disease, Abdominal Aortic Aneurysm	65	M	Caucasian
AAA 5	Aortic Disease, Abdominal Aortic Aneurysm	72	M	Caucasian
AAA 6	Aortic Disease, Abdominal Aortic Aneurysm	65	M	Caucasian
AAA 7	Aortic Disease, Abdominal Aortic Aneurysm	50	M	African American
AAA 8	Aortic Disease, Abdominal Aortic Aneurysm	31	M	Caucasian
AAA 9	Aortic Disease, Abdominal Aortic Aneurysm	71	M	American Indian or Alaska Native/Caucasian
